# New Report of Cyanobacteria and Cyanotoxins in El Pañe Reservoir: A Threat for Water Quality in High-Andean Sources from PERU

**DOI:** 10.3390/toxins16090378

**Published:** 2024-08-28

**Authors:** Victor Hugo Rodriguez Uro, Joana Azevedo, Mário Jorge Araújo, Raquel Silva, Jürgen Bedoya, Betty Paredes, Cesar Ranilla, Vitor Vasconcelos, Alexandre Campos

**Affiliations:** 1 Científica Peruana SRL., Calle Tupac Amaru 511, Mariano Melgar, Arequipa 04006, Peru; vrodriguez@cientificaperuana.com (V.H.R.U.); jurgenbedoya@gmail.com (J.B.); 2 Interdisciplinary Centre of Marine and Environmental Research (CIIMAR/CIMAR-LA), Terminal de Cruzeiros do Porto de Leixões, Av. General Norton de Matos, s/n, 4450-208 Porto, Portugal; joana.azevedo@ciimar.up.pt (J.A.); mario.araujo@ciimar.up.pt (M.J.A.); rssilva@ciimar.up.pt (R.S.); vmvascon@fc.up.pt (V.V.); 3 Departamento Académico de Química, Facultad de Ciencias Naturales y Formales, Universidad Nacional de San Agustín de Arequipa, Av. Independencia, s/n, Arequipa 04001, Peru; tparedesz@unsa.edu.pe; 4 Departamento Académico de Biología, Facultad de Ciencias Biológicas, Universidad Nacional de San Agustín de Arequipa, Av. Daniel Alcides Carrión, Arequipa 04002, Peru; cranilla@unsa.edu.pe; 5 Department of Biology, Faculty of Sciences, University of Porto, Rua do Campo Alegre, 4169-007 Porto, Portugal

**Keywords:** cyanobacteria, cyanotoxins, El Pañe high-Andean reservoir, PCR, LC-MS/MS

## Abstract

Cyanobacteria are cosmopolitan organisms; nonetheless, climate change and eutrophication are increasing the occurrence of cyanobacteria blooms (cyanoblooms), thereby raising the risk of cyanotoxins in water sources used for drinking, agriculture, and livestock. This study aimed to determine the presence of cyanobacteria, including toxigenic cyanobacteria and the occurrence of cyanotoxins in the El Pañe reservoir located in the high-Andean region, Arequipa, Peru, to support water quality management. The study included morphological observation of cyanobacteria, molecular determination of cyanobacteria (16S rRNA analysis), and analysis of cyanotoxins encoding genes (*mcyA* for microcystins, *cyrJ* for cylindrospermopsins, *sxtl* for saxitoxins, and *AnaC* for anatoxins). In parallel, chemical analysis using Liquid Chromatography coupled with Mass Spectrometry (LC-MS/MS) was performed to detect the presence of cyanotoxins (microcystins, cylindrospermopsin, saxitoxin, and anatoxin, among others) and quantification of Microcystin-LR. Morphological data show the presence of *Dolichospermum* sp., which was confirmed by molecular analysis. *Microcystis* sp. was also detected through 16S rRNA analysis and the presence of *mcyA* gene related to microcystin production was found in both cyanobacteria. Furthermore, microcystin-LR and demethylated microcystin-LR were identified by chemical analysis. The highest concentrations of microcystin-LR were 40.60 and 25.18 µg/L, in May and November 2022, respectively. Microcystins were detected in cyanobacteria biomass. In contrast, toxins in water (dissolved) were not detected. Microcystin concentrations exceeded many times the values established in Peruvian regulation and the World Health Organization (WHO) in water intended for human consumption (1 µg/L). This first comprehensive report integrates morphological, molecular, and chemical data and confirms the presence of two toxigenic cyanobacteria and the presence of microcystins in El Pañe reservoir. This work points out the need to implement continuous monitoring of cyanobacteria and cyanotoxins in the reservoir and effective water management measures to protect the human population from exposure to these contaminants.

## 1. Introduction

Cyanobacteria are prokaryotic organisms of cosmopolitan distribution. Their unique morphology and physiology provide several advantages in the colonization of a vast range of environments, in comparison to other microorganisms and green algae, which is evident through their capacity to grow rapidly and produce dense mats or blooms all over the world [1,2]. Cyanobacteria are considered an emerging threat to freshwater resources [3]. The cyanoblooms are defined as biomass increments of cyanobacteria mainly caused by one or a few species in a short period of time [4]. The classification of cyanobacteria is commonly performed following morphological traits. However, with polyphasic classification, the morphological identification must be complemented with molecular identification and ecological information [5]. Nowadays, molecular biology tools serve to study and monitor toxigenic cyanobacteria and involve the detection and identification of cyanobacteria as well as the genes related to the production of cyanotoxins [6].

Cyanotoxins are secondary metabolites produced by cyanobacteria; they have potent biological activity and can cause adverse effects on humans, terrestrial animals, aquatic organisms, and the environment [7]. The most common and better-studied cyanotoxins are microcystins, cylindrospermopsins, anatoxins, saxitoxins, and anatoxin-a(S) [8,9]. Based on their toxicological target cyanotoxins can be classified as hepatotoxins, cytotoxins, neurotoxins, and dermatoxins. Furthermore, microcystins are the most prevalent toxins in freshwater and are also among the potent cyanotoxins. Microcystins belong to the group of hepatotoxins and are cyclic heptapeptides composed by D–alanine at position 1, two variable L–amino acids at positions 2 and 4, γ–linked D–glutamic acid at position 6, and 3 unusual amino acids; β–linked D–erythro–β–methylaspartic acid (MeAsp) at position 3; and (2S, 3S, 8S, 9S)-3-amino-9-methoxy– 2, 6, 8 trimethyl–10-phenyldeca–4 and 6–dienoic acid (Adda) at position 5 and N–methyl dehydroalanine (MDha) at position 7 [10]. Several episodes of human poisoning have been reported, the most known occurred in Brazil, where 56 patients died at a hospital after hemodialysis treatment with water accidentally contaminated with microcystins [7]. Furthermore, wild animals and livestock are frequently poisoned by consuming water contaminated with toxic cyanobacteria [9,11]. Cyanotoxins pose a potential risk to human health and the environment and in consequence, the World Health Organization (WHO) established reference limit values for cyanotoxins in drinking and recreational waters [12,13].

El Pañe reservoir is one of the seven high-Andean water reservoirs in the hydrographic basin of Chili River. This reservoir was built in 1975 by flooding three small lakes and is located at 4580 m above sea level (m.a.s.l.) in the Peruvian Andean plateau. In this region, the environmental conditions are extreme and characterized by lower levels of oxygen, low barometric pressure, high solar and UV radiation, low rates of precipitation, high rates of evaporation, strong winds, and a wide diurnal temperature range [14]. This reservoir is one of the main water sources for drinking water, agriculture, and livestock in the Arequipa region, Peru. Furthermore, the reservoir holds fish farms developed by local people [15,16]. Fish production is a source of nutrients to the reservoir (fish feed and fish feces), contributing to the eutrophication of the aquatic media [17]. Since 2014, the water management authorities have been reporting the presence of cyanobacteria and cyanoblooms in El Pañe reservoir [17,18].

Taking into account the eutrophication issues of El Pañe reservoir, this work aimed to determine the presence of cyanobacteria and cyanotoxins from water samples collected in the year 2022. The study combined morphological, molecular, and chemical analyses and revealed the presence of toxic cyanobacteria and microcystins above the regulatory levels in the reservoir. This comprises the first scientific report on cyanobacteria from the Peruvian Andean plateau.

## 2. Results

### 2.1. Morphological Analysis

**Figure 1:**
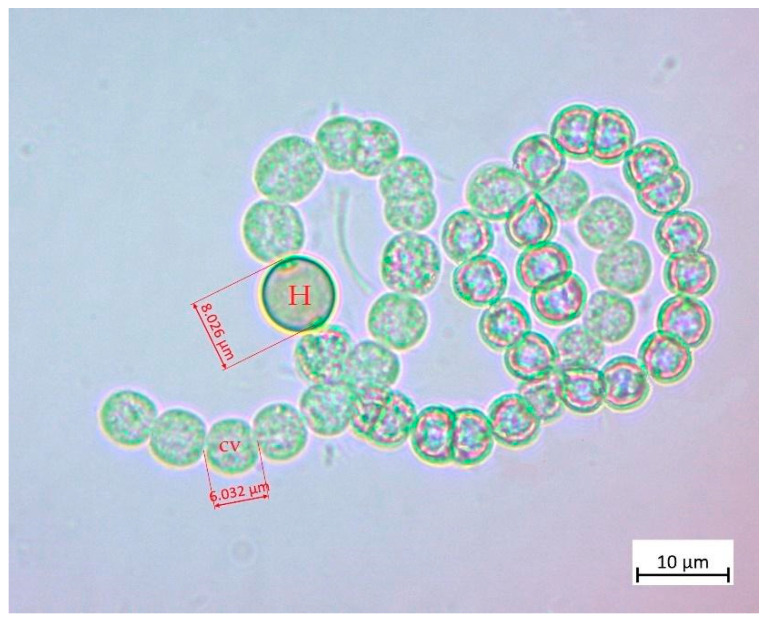
Microphotograph at 100× magnification of genus *Dolichospermum* sp. from El Pañe reservoir displaying heterocytes (H) and vegetative cells (CV).

Based on taxonomic keys [19], the morphospecies found in El Pañe reservoir belongs to the genus *Dolichospermum* sp. This morphospecies was found in all sampling points in the two sampling campaigns (May and November 2022). The genus *Dolichospermum* sp. is planktonic and, according to the morphological traits observed, develops gas vesicle aggregates in aerotopes. This genus is usually present as solitary filaments and occasionally in clusters and fascicules. Filaments can be straight, coiled, or screw-like contorted, with isopolar trichomes and specialized cells (heterocytes and akinetes). Cells are approximately isodiametric or longer than wide, with barrel-shape or spheroidal, and their diameter is wider than 2.5–3 µm, as shown in Figure 1.

### 2.2. Molecular Analysis of Cyanobacteria Genes

#### 2.2.1. Molecular Analysis of Cyanobacteria 16S rRNA Genes

Regarding the results about the presence of cyanobacteria in El Pañe, we can confirm the presence of cyanobacteria in all the analyzed samples (Table 1). Using specific primers for cyanobacteria 16S rRNA genes, *Microcystis* sp. 16S rRNA genes, and *Dolichospermum* sp 16S rRNA genes, we report the amplification of these gene sequences, confirming the presence of the genus *Microcystis* sp. in only one sample from November 2022 (sample code–EPP2N) (Supplementary Figure S1) and the genus *Dolichospermum* sp. in all the samples from May and November 2022 (Table 1).

#### 2.2.2. Molecular Analysis of Cyanotoxin-Encoding Genes

In Table 2 we report the molecular analysis results regarding the detection of cyanotoxins-encoding genes. Microcystin-encoding gene (*mcyA*) was detected in all the samples analyzed by gel electrophoresis of the PCR products (Supplementary Figure S2), indicating the presence of putative microcystin-producing cyanobacteria. However, gene sequences corresponding to *cyrJ*, *anaC*, and *sxtl* were not detected, which suggests the absence of cyanobacteria producing cylindrospermopsins, anatoxins, and saxitoxins.

#### 2.2.3. Molecular Analysis of Microcystin-Encoding Genes from *Microcystis* sp.

The genes *mcyABCDE*, from the microcystin-encoding gene-cluster in *Microcystis* sp., were analyzed. The results are shown in Table 3. Sample 2 from November 2022 (Sample code EPP2N) was the only sample where amplification of the genes *mcyBCDE*-*Microcystis* sp. was obtained. Furthermore, the gene *mcyA*-*Microcystis* sp. was not detected in any of the samples.

### 2.3. Chemical Analysis of Cyanotoxins

#### 2.3.1. Cyanotoxins Analysis

**Figure 2:**
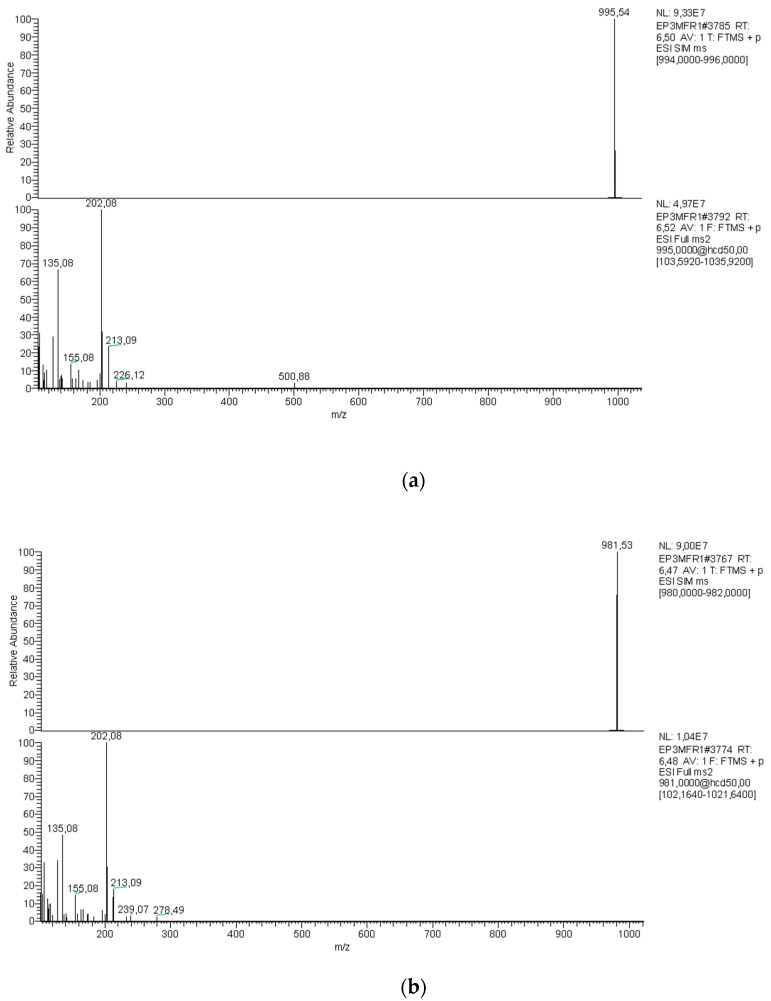
Mass spectrum for the sample EPP3M from El Pañe reservoir: single ion monitoring and MS^2^ of (**a**) MC-LR (*m*/*z* 995.54) and daughter ion peaks (135.08 and 213.09 *m*/*z*) and (**b**) demethylated MC-LR ([Dasp^3^]MC-LR, [Dha^7^]MC-LR) (*m*/*z* 981.53) and daughter ion peaks (135.08 and 213.09 *m*/*z*). Abbreviation: *m*/*z*, mass-to-charge ratio.

The LC-MS/MS analysis in full scan mode allowed the detection of microcystin-LR (MC-LR). Subsequently, analysis in the multiple reaction monitoring (MRM) mode confirmed the presence of MC-LR in the phytoplankton biomass collected after filtering the water sample, but not in the filtered water (dissolved microcystin). This analysis is based on the detection of the parent ion peak corresponding to MC-LR ([M+H]^+^ *m*/*z* 995.54) and the diagnostic fragmentation product (*m*/*z* 135.5) (Figure 2a). In addition, we found a demethylated MC-LR, ([Dasp^3^]MC-LR, [Dha^7^]MC-LR), corresponding to the parent ion peak (*m*/*z* 981.5) and fragmentation products (*m*/*z* 135.08 and 213.09) (Figure 2b).

#### 2.3.2. Microcystin Quantification

The results show important amounts of MC-LR in biomass (cells). The highest concentrations were found in samples EPP3M and EPP3N, which are, respectively, 25.18 µg/L and 40.60 µg/L MC-LR (Table 4). Additionally, a relevant concentration (12.3 µg/L) of demethylated MC-LR was registered in sample EPP3N ([Dasp^3^]MC-LR, [Dha^7^]MC-LR).

## 3. Discussion

El Pañe reservoir is an important source of water, providing drinking water for a densely populated region (Arequipa) and water for agriculture, livestock, and aquaculture activities. However, this reservoir is located in a remote area with difficult access to sampling and fieldwork. To understand the presence of cyanobacteria in El Pañe, it is important to consider its elevation of 4580 m.a.s.l., situated in the Peruvian Andean plateau. This region endures extreme climate conditions, with dry winters and rainy summers. [15,20]. The annual mean temperature is 7 °C, with winter temperatures averaging 1 °C, summer temperatures averaging 6 °C, and a wide diurnal temperature fluctuation [16,20]. The samples for this study were taken in May and November corresponding to the dry and rainy seasons with a great influence on the limnology of the reservoir. The water temperatures registered for these samples were 9.5 °C and 12 °C, respectively. Furthermore, the water temperature in El Pañe is higher than the air average temperature. In 2018, for instance, the lowest water temperature was 7.97 °C and the highest was 14.34 °C [15]. Cyanoblooms are usually favored in high temperatures above 25 °C [13]. However, these organisms are known to colonize and proliferate in adverse environments, having developed physiological strategies that enable their survival and growth [13,21]. Another aspect to consider is the trophic state of the reservoir. The literature reports signs of eutrophication in El Pañe reservoir since 2014 [17]. Although physicochemical data obtained in this study regarding total phosphorus, total nitrogen, and chlorophyll (Supplementary Table S5) do not evidence eutrophication in the reservoir, aquaculture and livestock are two activities that are contributing to nutrient load in the reservoir [22].

There are few studies reporting cyanobacteria in this region of Peru. Reports of cyanobacteria occurrences have been documented in geographically close regions, including Puno Bay (Lake Titicaca), where it was identified as potentially bloom-forming cyanobacteria from genera *Microcystis*, *Nodularia*, and *Limnoraphis* [23,24]. In the present study, we complement the existing records of cyanobacteria in the Andean plateau by reporting the presence of the genus *Dolichospermum* sp. and *Microcystis* sp. employing microscopic and molecular analyses. The presence of the genus *Dolichospermum* sp. is consistent with previous review of planktonic cyanobacteria in Peruvian freshwater [18]. The occurrence of this genus and also *Microcystis* sp. in this habitat is not surprising as both show wide geographical distribution (sub-tropical and temperate regions) and can be found in many different habitats and tolerate cold water [7,25]. Capelli et al. (2017) [26] highlight the ecological heterogeneity of the species *Dolichospermum lemmermannii* and raise the hypothesis of the existence of different ecotypes and phylogenetic lineages driven by geographic isolation, physical barriers, and environmental factors. Thus, it is most likely that the *Dolichospermum* sp. identified in the El Pañe reservoir belongs to an ecotype adapted to this specific environment.

Molecular analysis was important to conduct this study. Indeed, the genus *Microcystis* sp. was not detected by microscopy, instead using a molecular method based on 16S rRNA markers. Furthermore, the amplification of genes related to the production of microcystins (*mcyA*), saxitoxins (*sxtl*), cylindrospermopsins (*cyrJ*), and anatoxins (*anaC*) was performed. All samples from El Pañe reservoir were positive with respect to the presence of the *mcyA* gene, one of the 11 genes present in the microcystin-encoding gene cluster. The primer used in this analysis was designed to amplify the *mcyA* gene from several cyanobacteria genera. However, when the analysis with specific primers for *mcyABCDE* genes for *Microcystis* sp. was performed, it revealed the presence of *mcyBCDE* genes only in the sample EPP3 corresponding to the month of November. This sample was the only one where we detected the presence of *Microcystis* sp. using a 16S rRNA marker. In this analysis, the *Microcystis* sp. *mcyA* gene was not identified. Possible causes for the absence of amplification of this gene are the occurrence of mutations in the gene cluster [27,28]. The results indicate that the cyanobacteria *Microcystis* sp. and *Dolichospermum* sp. identified are both potential producers of microcystins. The results also are in accordance with the literature that indicates that the genus *Dolichospermum* is able to produce microcystins, saxitoxins, and anatoxins [4,29] and the genus *Microcystis* can produce microcystins and anatoxins [4,9,13]. On the other hand, molecular analysis of the genes related to the production of saxitoxins, cylindrospermopsins, and anatoxins could not detect any of those genes, meaning that the cyanobacteria in El Pañe reservoir are unlikely to produce other cyanotoxins. We also may expect *Dolichospermum* sp. to occur all year round in the reservoir, whereas *Microcystis* sp. might be more influenced by water temperature and seems to occur only during the warmer period (rainy season). Indeed, previous studies evidence the dominance of the genus *Dolichospermum* sp. in the reservoir as a potentially toxic cyanobacteria [17,18].

Knowing that molecular analysis of cyanotoxins-encoding genes will not confirm the presence of cyanotoxins in the reservoir, we also performed a chemical analysis of the water. Biosynthesis of cyanotoxins is a complex process, involving multiple enzymes/genes and regulatory mechanisms. Often, strains holding complete biosynthetic gene clusters do not synthetize toxins [30]. For this reason, chemical analysis is crucial to infer the presence of cyanotoxins in the water. In this work, we report the presence of microcystins and quantify MC-LR and demethylated MC-LR by LC-MS/MS. Other toxins such as cylindrospermopsins, saxitoxins, and anatoxin-a seem to be absent in the reservoir. This result is coherent with the results obtained regarding the toxin-encoding genes. Regarding microcystins, two chemical variants were identified MC-LR (*m*/*z* 995.54) in biomass samples (cells) and demethylated microcystins (*m*/*z* 981.53) corresponding to [Dasp^3^]MC-LR and [Dha^7^]MC-LR [28]. Quantification of MC-LR dissolved in water was not possible, the toxin was below the detection limit of the method. On the other hand, quantification of MC-LR in biomass samples (intracellular MC-LR) showed high concentrations achieving 25.18 µg/L and 40.60 µg/L in May and November, respectively. These samples were collected close to fish cages where the cyanobloom is more intense and cell density is higher. Unfortunately, cell density was not determined during microscopy investigations, impeding to compare and relate cell density and toxin concentration. The capability of the genus *Dolichospermum* sp. to produce microcystins and the prevalence of intracellular microcystins in *Dolichospermum* sp. blooms have been previously described in the literature [9,29]. These results are also consistent with the numerous reports confirming that microcystins are preferentially accumulated in cyanobacteria cells [9]. Toxin release to the water occurs under specific conditions when cells lyse and die [8]. To our knowledge, microcystins and anatoxin-a in the El Pañe reservoir were reported only once [31]. Regarding anatoxin-a, we cannot confirm the occurrence of this toxin. Taking the present results, a continuous and systematic characterization of cyanobacteria communities and cyanotoxins in the reservoir is still necessary to further infer the causes of their occurrence and prevalence.

The high concentrations of microcystins reported in this study raise water management concerns in El Pañe. In Peruvian regulation (No. 004–2017-MINAM), only two parameters are considered in the assessment of water quality: 1: the number of free-living organisms and 2: the MC-LR concentration. Free-living organisms comprise all algae, protozoa, copepods, rotifers, and nematodes in all their living stages and the value should not exceed 5 × 10^6^ organisms per liter in water intended for human consumption. However, there is no specific national regulation regarding the identification and quantification of cyanobacteria or any reference to biovolume. In the case of microcystins, the regulation establishes that the MC-LR concentration in water intended for human consumption should not exceed 1 µg/L. The national regulation is consistent with the WHO guidelines for drinking water. The WHO also proposes a limit concentration of 12 µg/L microcystin-LR in drinking water, considering short-term exposure and for recreational water of 24 µg/L microcystin-LR. In this work, we report values of MC-LR 40-fold higher and more than 52-fold higher (52.90 µg/L) considering also demethylated microcystins. The recommendation followed in many countries is the quantification of total microcystins and not only microcystin-LR, for the assessment of water contamination. Since the water from El Pañe intended for human consumption is currently subject to treatment for removal of different contaminants including cyanobacteria and cyanotoxins and properly monitored regarding these contaminants, the main risks we identify in the use of El Pañe water concern livestock poisoning and fish contamination. There is also a risk of human exposure by consuming contaminated fish produced in the reservoir. We also point out that other reservoirs in this region could show similar problems of contamination with cyanobacteria and, therefore, it would be important to extend the monitoring work to those water bodies as well. To ensure effective water quality management in the region of Arequipa, reducing and mitigating the risk to human health and the environment, it is imperative to conduct systematic studies of cyanobacteria and cyanotoxins and implement adequate cyanobacteria monitoring programs.

## 4. Conclusions

This work is the first report integrating morphological, molecular, and chemical approaches to report the presence of cyanobacteria and cyanotoxins in El Pañe reservoir in two distinct seasons. We report the presence of cyanobacteria, specifically the genera *Dolichospermum* sp. and *Microcystis* sp., through morphological traits and molecular methods. Additionally, the *mcyA* gene involved in the synthesis of microcystins was detected in both cyanobacteria. Chemical analysis through LC-MS/MS confirmed the presence of two microcystins, MC-LR and demethylated MC-LR ([Dasp^3^]MC-LR and [Dha^7^]MC-LR). The highest concentrations found for MC-LR were 25.18 µg/L and 40.60 µg/L in samples from May and November, respectively. Furthermore, an important concentration of demethylated MC-LR was reported for the sample from November, 12.30 µg/L. These values exceed Peruvian water quality legislation and the values proposed by WHO many times. This report is valuable for governmental institutions and the population, serving as a valuable resource to manage the associated risks of cyanobacterial blooms and toxin exposure and implement targeted interventions to safeguard both human health and the environment.

## 5. Materials and Methods

### 5.1. Sampling

**Figure 3:**
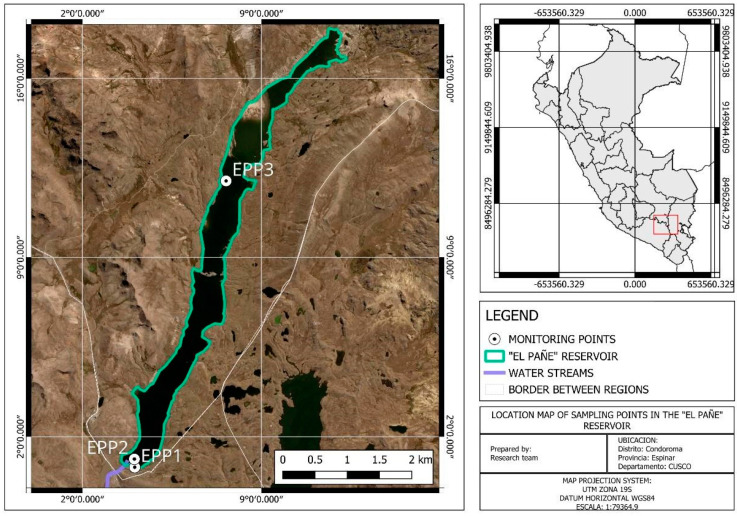
Distribution map of the sampling points and the localization of El Pañe reservoir.

The sampling location was El Pañe reservoir, Arequipa region—Peru, and the geographic coordinates are shown in Table 5. Two sampling campaigns were conducted during the year 2022, in May and November corresponding to the dry and rainy seasons, respectively. Three sampling points were established: 2 downstream near the dam and another in the middle part of the reservoir near the aquaculture cages (Figure 3). All the 20 samples collected correspond to individual samples. Each sample was collected at the water surface in one polyethylene bottle of 200 mL and two polyethylene bottles of 1 L. For morphological identification, the samples were collected using a nylon phytoplankton net (20 µm pore size), transferred to a bottle of 200 mL, and preserved with Lugol’s iodine solution. The samples for molecular biology and cyanotoxins analyses were collected directly from the water surface to 1L bottles. All the samples were preserved under cold conditions until arriving at the laboratory.

### 5.2. Field and Laboratory Analyses

Field analyses (temperature, pH, conductivity, total dissolved solids, dissolved oxygen, and turbidity) were performed in situ using a portable multiparameter (Hanna Instruments 9829, Romany) (Supplementary Table S5). Laboratory analyses performed are listed in Table 6.

#### 5.2.1. Microscopy Observation

All the samples collected for morphological analysis were observed using an optical microscope Panthera C2 (Motic, Hong Kong, China) integrated with a camera Moticam S6 (Motic, Xiamen, China). The magnification used was 10×, 40×, and 100×. The morphological classification was carried out according to the morphological traits described in the literature [5,19].

#### 5.2.2. Molecular Analysis

For the molecular biology analysis, the water samples were filtered using a vacuum filtration system (Rocker 300, Taiwan) and 47 mm diameter glass filters (1 µm pore size, Pall, Port Washington, NY, USA). Subsequently, the filters were lyophilized and stored at −20 °C until further processing.

The lyophilized samples were then scraped using a sterile scalpel and placed into 2 mL test tubes. Genomic DNA (gDNA) from the samples were extracted using the PureLink™ Genomic DNA Mini Kit (Invitrogen, Waltham, MA, USA), following the protocol for Gram-negative bacteria. A volume of 30 µL of elution solution was used for each extracted DNA sample. To confirm the presence of DNA, electrophoresis in agarose gel (1% *w*/*v*) (UltraPure™ Agarose, Invitrogen, Carlsbad, CA, USA) was performed, using a buffer solution of 40 mM Tris-Acetate, 1 mM EDTA, pH 8.3 ± 0.10, and SYBR™ Safe DNA Gel Stain (Invitrogen, CA, USA). Electrophoresis was conducted at a constant voltage of 100 V for 22 min. In each well of the electrophoresis gel, gDNA (2 µL) was loaded with 1 µL of 1× gel loading buffer consisting of 10 mM Tris-HCl, pH 8, 5% glycerol, 1 mM EDTA, 0.04% bromophenol blue, and 0.04% xylene cyanol FF. A Gel Doc™ EZ Imager (Bio-Rad, Hercules, CA, USA) with UV light was used to visualize the results of the electrophoresis run. The obtained images were viewed and exported using ImageLab 6.1 software (Bio-Rad, USA).

For DNA amplification, the endpoint PCR technique was followed, with a final volume of 20 µL. Master mix solutions were prepared for each DNA segment of interest, taking into account the following quantities: 4 µL of 5x Green GoTaq^®^ Flexi buffer, 2 µL of 25 mM MgCl_2_ solution, 2 µL of forward primer, 2 µL of reverse primer, 1.5 µL of 2.5 mM mixture of deoxynucleotide triphosphates (dNTPs), 0.5 µL of bovine serum albumin (BSA) (10 mg/mL), 0.1 µL of GoTaq^®^ DNA Polymerase (5 U/μL), and 1 µL of DNA sample. PCR reactions were carried out in a Veriti™ 96-Well Thermal Cycler (Applied Biosystems, Foster City, CA, USA). Electrophoresis to confirm the PCR results was performed in agarose gel (1.5% *w*/*v*).

##### Molecular Analysis of Cyanobacteria

To confirm the presence of cyanobacterial DNA from the extracted DNA of environmental water samples, PCR amplification was performed targeting 16S ribosomal RNA (rRNA) fragments. The primer pairs used were 27F/1494R and CYA359F/CYA781R for the 16S rRNA genes. Specific primers for *Microcystis* sp. and *Dolichospermum* sp. were also used. The primers used for *Microcystis* sp. were Micr 184F and Micr 431R, while for *Dolichospermum* sp., ANA 573F and ANA 780R were employed (Tables S1 and S2).

##### Molecular Analysis of Cyanotoxins-Encoding Genes

After confirming the presence of cyanobacterial DNA, the amplification of genes encoding cyanotoxins was performed in all samples. The amplified genes were *mcyA* for microcystins, *cyrJ* for cylindrospermopsins, *sxtl* for saxitoxins, and *AnaC* for anatoxins. Primers and PCR conditions are described in Supplementary Tables S1 and S2.

As a positive control for the amplification reactions (PCR), DNA extracted from pure cultures of strains belonging to LEGE Culture Collection (LEGE-CC, https://lege.ciimar.up.pt) was used. To determine the presence of genes producing microcystins, cylindrospermopsins, saxitoxins, and anatoxins, the strains LEGE-00063, LEGE-97047, LMECYA-031, and LEGE-X002 were utilized, respectively.

##### Molecular Analysis of Microcystin-Encoding Genes from *Microcystis* sp.

To determine the presence of microcystin-encoding genes in *Microcystis* sp., specific primers targeting the *mcy* gene cluster were used, including *mcyA*, *mcyB*, *mcyC*, *mcyD*, and *mcyE* (Tables S1 and S2).

#### 5.2.3. Chemical Analysis of Cyanotoxins

To determine the content of cyanotoxins, 1 L water samples were filtered, using a vacuum system of filtration (Rocker 300, Taiwan) and 47mm diameter glass filters (1 µm pore size, Pall, USA) separating cyanobacteria cells (biomass) and water. This separation was performed separately to determine the cyanotoxins associated with cyanobacteria cells biomass (intracellular) and the cyanotoxins dissolved in the water (extracellular).

##### Extraction

The extraction of cyanotoxins from filtered water was carried out using a 12-port SPE vacuum manifold and SPE cartridges C18 (Oasis ^®^ HLB, 200mg, 6cc). The cartridges were first conditioned with 5 mL of methanol analytic grade (MeOH) and 5 mL distilled water (H_2_O). The samples passed through the cartridges with a flow (>1 mL/min). Elution was performed with 5 mL of MeOH (80 *v*/*v*) and collected in 15 mL volume glass vials. The eluted volume was evaporated using a rotavapor R-300 (Buchi, Flawil, Switzerland) and the residue was resuspended with 500 µL of a solution of 50% MeOH (LC/MS grade) acidified with 0.1% formic acid (FA) and transferred to 2 mL HPLC vial. Samples were stored at −20 °C until LC-MS/MS analysis [32].

To extract the cyanotoxins from cyanobacteria cells collected in filters, these filters were first lyophilized and subsequently ground in a lab mortar with liquid nitrogen. Immediately afterward, 5 mL of a solution of 10% MeOH was added and transferred to a 15 mL falcon tube. The samples were sonicated in an ice bath (5 times × 1 min, 50%) using a sonicator Sonoplus HD-2070-2 (Bandelin, Berlin, Germany) and centrifuged at 10,000× *g* for 20 min in a centrifuge Megafuge 16R (Thermo Scientific, Waltham, MA, USA). The supernatant was collected in glass tubes and the solvent was evaporated using a rotavapor R-300 (Buchi, Switzerland). The dry extract was resuspended with 1 mL of a solution of 50% MeOH (LC/MS grade) acidified with 0.1% formic acid (FA) and transferred to a 2 mL HPLC vial. Samples were stored at −20 °C until LC-MS/MS analysis.

##### LC-MS/MS Analysis

The LC-MS/MS system used to determine cyanotoxins was a Liquid Phase Chromatograph Alliance e2695 (Waters) coupled with a triple quadrupole mass spectrometer (Micromass^®^ Quattro micro-TM API) equipped with electrospray ionization (ESI) interface. The software used for data processing was MassLynx version 4.1. The mass spectrometer was operated in full scan mode and multiple reaction monitoring (MRM). The conditions were the following: the capillary voltage was maintained at 3.5 kV, the cone voltage at 20 V, the extractor at 3 V, and the lens at 0.2 V. The source temperature was set at 120 °C, while the desolvation temperature was maintained at 350 °C with a desolvation gas flow rate of 500 L/h. Nitrogen gas was used as the sheath gas and auxiliary gas, while argon gas served as the collision gas at a pressure of 0.5 bar. The separation of compounds was carried out on a C18 Hypersil Gold column (100 × 4.6 mm I.D., 5 μm, ThermoScientific, Waltham, MA, USA) maintained at a temperature of 45 °C, with a flow rate of 0.50 mL/min. A volume of 10 µL was injected using a partial loop mode. The samples were injected in positive polarity under full scan conditions (30–2000 *m*/*z*). The standards and samples were injected in duplicate and a blank and two standards of different concentrations were introduced at each set of 10 samples. An elution gradient was employed using the following mobile phases: solvent A, MeOH, and solvent B, H_2_O, both LC-MS grade and acidified with 0.1% formic acid. The gradient conditions were as follows: starting at 10% A and 90% B for 3 min, transitioning to 40% A in 1 min, increasing to 60% A in 7 min (held for 2 min), increasing to 80% A for 2 min, and returning to initial conditions at 20 min (held for 10 min).

The standard solutions and quality control solutions (extracts of cyanobacteria toxin producers) used for the optimization of mass parameters were as follows: anatoxin (CRM-03-ANA, Lot 16-001, purity: 99%; saxitoxin (CRM-00-STX, Lot 16-001, purity: 99%) and cylindrospermopsin (CRM-03-CYN, Lot 16-001, purity: 99%). These three standards were provided by CIFGA (Lugo, Spain). A Hepatotox SetTM 1, containing MC-LR, MC-RR, MC-YR, MC-LW, MC-LF, and NOD-R, was supplied by ALEXIS Biochemicals (Boston, MA, USA, Lot L26789, >95% purity). As mentioned previously, the quantification of the samples was carried out in MRM mode, using the transitions specified in Table S3.

The LOD and LOQ of the instrument were determined by regression analysis [33].

##### Microcystins Quantification

Estimation of the content of microcystin-LR was performed using an external standard. The MC-LR standard solution used for calibration had a concentration of 10 µg/L and was purchased from CIFGA S.A. (Spain). The system conditions and the injection conditions are the same as described above and the quantification of MC-LR was carried out using MRM mode.

Only two samples (EPP2N and EPP3N) were analyzed by an external laboratory (CIFGA S.A.). The analysis conditions are shown in Supplementary Table S4.

## Figures and Tables

**Table 1:** Molecular analysis of cyanobacteria from El Pañe reservoir.

Code	Cyanobacteria	*Microcystis* sp.	*Dolichospermum* sp.
	16S rRNA	16S rRNA *Microcystis* sp.	16S rRNA *Dolichospermum* sp.
EPP2M	+	−	+
EPP3M	+	−	+
EPP2N	+	+	+
EPP3N	+	−	+

(+) Gene detected and (−) gene not detected by PCR.

**Table 2:** Molecular analysis of cyanotoxins-encoding genes from El Pañe reservoir.

Code	Microcystins	Cylindrospermopsins	Anatoxins	Saxitoxins
	*mcyA*	*cyrJ*	*AnaC*	*Sxtl*
EPP2M	+	−	−	−
EPP3M	+	−	−	−
EPP2N	+	−	−	−
EPP3N	+	−	−	−

(+) Gene detected and (−) gene not detected by PCR.

**Table 3:** Molecular analysis of microcystins-encoding genes from *Microcystis* sp. in El Pañe reservoir.

Code	Microcystins *Microcystis* sp.				
	*mcyA*- *Microcystis* sp.	*mcyB*- *Microcystis* sp.	*mcyC*- *Microcystis* sp.	*mcyD*- *Microcystis* sp.	*mcyE*- *Microcystis* sp.
EPP2M	−	−	−	−	−
EPP3M	−	−	−	−	−
EPP2N	−	+	+	+	+
EPP3N	−	−	−	−	−

(+) Gene detected and (−) gene not detected by PCR.

**Table 4:** Content of microcystin-LR in samples from El Pañe reservoir.

Code	Origin	Month	Results			
			Units	MC-LR Biomass	dmMC-LR ^1^ Biomass	MC-LR Dissolved
EPP1M	El Pañe	May	µg/L	2.17	-	<LOD
EPP2M	El Pañe	May	µg/L	19.39	-	<LOD
EPP3M	El Pañe	May	µg/L	25.18	-	<LOD
EPP1N	El Pañe	November	µg/L	-	-	-
EPP2N	El Pañe	November	µg/L	0.46	0.11	0.14
EPP3N	El Pañe	November	µg/L	40.60	12.3	<LOD

^1^ dmMC-LR: Demethylated MC-LR; LOD: Limit of Detection; (-) analysis not performed.

**Table 5:** Geographic coordinates of sampling points in El Pañe reservoir.

Code	Origin	Coordinates	
		GPS Lat.	GPS Long.
EPP1	El Pañe	15°25′07.6″ S	71°04′01.8″ W
EPP2	El Pañe	15°25′07.6″ S	71°04′01.9″ W
EPP3	El Pañe	15°20′19.6″ S	71°02′23.8″ W

**Table 6:** Samples were collected from El Pañe reservoir and analyses were performed.

Code	Origin	Month	Analysis			
			Microscopy	Molecular Biology	Chemistry Biomass	Chemistry Water
EPP1M	El Pañe	May	x		x	x
EPP2M	El Pañe	May	x	x	x	x
EPP3M	El Pañe	May	x	x	x	x
EPP1N	El Pañe	November	x			
EPP2N	El Pañe	November	x	x	x	x
EPP3N	El Pañe	November	x	x	x	x

## Data Availability

Data are provided in the article.

## Supplementary Materials

The following supporting information can be downloaded at https://www.mdpi.com/article/10.3390/toxins16090378/s1: Table S1: PCR Primers and conditions for the molecular identification of cyanobacterial strains and cyanotoxins genes.; Table S2: Names and sequences of primers used to perform biology molecular analysis.; Table S3: Cyanotoxins, parameters for MS analysis and operation, and instrument limit of detection (LOD); Table S4: Microcystins analyses conditions carried out by external laboratory CIFGA S. A.; Table S5: Field parameters in water samples from El Pañe reservoir; Figure S1: Amplification of 16S rRNA gene specific for *Microcystis* sp. in samples from El Pañe reservoir.; Figure S2: Amplification of microcystin encoding-gene (*mcyA*) in samples from El Pañe reservoir. References [34,35,36,37,38,39,40,41,42,43] are cited in the Supplementary Materials.
