# Supplementary material for: New Report of Cyanobacteria and Cyanotoxins in El Pañe Reservoir: A Threat for Water Quality in High-Andean Sources from PERU
Source: Toxins (Basel). 2024 Aug 28;16(9):378. doi: 10.3390/toxins16090378 (PMC11435830; doi:10.3390/toxins16090378)
Supplement: Supplementary file 1 [file toxins-16-00378-s001.zip › toxins-3128355-supplementary.pdf]

New Report of Cyanobacteria and Cyanotoxins in El Pañe Reservoir: A Threat for Water Quality in High-Andean Sources from PERU

Victor Hugo Rodriguez Uro, Joana Azevedo, Mário Jorge Araújo, Raquel Silva, Jürgen Bedoya, Betty Paredes, Cesar Ranilla, Vitor Vasconcelos and Alexandre Campos

Table S1. PCR Primers and conditions for the molecular identification of cyanobacterial strains and cyanotoxins genes.

Target Gene	Primers	PCR Conditions				Product size (bp)	Reference
		Step	Temperature	Time	N° cycles		
16S rRNA	27F/1494R	Initial denaturation	95°C	2min		754	[34]
	CYA359F/CYA781R	Denaturation	95°C	1min		1133	[35]
		Annealing	55°C	45s	35		
		Extension	72°C	1min			
		Final Extension	72°C	5min			
		Holding	8°C	Pause			
16S rRNA	Micr 184F	Initial denaturation	95°C	2 min		220	[34]
Microcystis sp.	Micr 431R	Denaturation	95°C	90s			
		Annealing	56°C	30s	35		
		Extension	72°C	50s			
		Final Extension	72°C	7min			
		Holding	8°C	Pause			
16S rRNA	Ana 573F	Initial denaturation	95°C	2 min			[36]
Dolichospermun sp.	Ana 780R	Denaturation	94°C	30s			

		Annealing	56°C	30s	35		
		Extension	72°C	1min			
		Final Extension	72°C	7min			
		Holding	8°C	Pause			
Microcystin<br>*mcy A*	mcyA_CD1F<br>mcyA_CD1R	Initial denaturation	95°C	2 min		297	[37]
		Denaturation	95°C	90s			
		Annealing	56°C	30s	35		
		Extension	72°C	50s			
		Final Extension	72°C	7min			
		Holding	8°C	Pause			
Microcystis sp.<br>*mcyA*	MSF<br>MSR	Initial denaturation	95°C	2 min		1300	[38]
		Denaturation	95°C	90s			
		Annealing	60°C	30s	35		
		Extension	72°C	50s			
		Final Extension	72°C	7 min			
		Holding	8°C	Pause			
Microcystis sp.<br>*mcyB*	2156-F<br>3111-R	Initial denaturation	94°C	5 min		955	[39]
		Denaturation	95°C	1 min			
		Annealing	52°C	30 s	35		
		Extension	72°C	1 min			
		Final Extension	72°C	7 min			
		Holding	8°C	Pause			
Microcystis sp.<br>*mcyC*	PSCF1<br>PSCR1	Initial denaturation	94°C	5 min		674	[40]
		Denaturation	95°C	1 min			

		Annealing	52°C	30 s	35		
		Extension	72°C	1 min			
		Final Extension	72°C	7 min			
		Holding	8°C	Pause			
Microcystis sp.	PKDF1	Initial denaturation	94°C	5 min		647	[40]
mcyD	PKDR1	Denaturation	95°C	1 min			
		Annealing	52°C	30 s	35		
		Extension	72°C	1 min			
		Final Extension	72°C	7 min			
		Holding	8°C	Pause			
Microcystis sp.	PKEF1	Initial denaturation	94°C	5 min		755	[40]
mcyE	PKER1	Denaturation	95°C	1 min			
		Annealing	52°C	30 s	35		
		Extension	72°C	1 min			
		Final Extension	72°C	7 min			
		Holding	8°C	Pause			
Cylindrospermopsin	cynsulF	Initial denaturation	95°C	3 min		584	[41]
cyrJ	cylnamR	Denaturation	95°C	45s			
		Annealing	50°C	30s	35		
		Extension	72°C	1 min			
		Final Extension	72°C	3 min			
		Holding	8°C	Pause			
Saxitoxin	sxtl682F	Initial denaturation	94°C	3 min		200	[42]
sxtl	sxtl877R	Denaturation	94°C	10 s			

		Annealing	52°C	20 s	35		
		Extension	72°C	1 min			
		Final Extension	72°C	7 min			
		Holding	8°C	Pause			
Anatoxin	anaC-gen F	Initial denaturation	94°C	2 min		366	[43]
AnaC gene	anaC-gen R	Denaturation	94°C	30 s			
		Annealing	58°C	30 s	35		
		Extension	72°C	30 s			
		Final Extension	72°C	5 min			
		Holding	8°C	Pause			

Table S2. Names and sequences of primers used to perform biology molecular analysis

Target gene	Primer name	Sequence
16S rRNA	27F	AGAGTTTGATCCTGGCTCAG
	1494R	TACGGCTACCTTGTTACGAC
	CYA359F	GGGGAATYTTCCGCAATGGG
	CYA781R	GACTACWGGGGTATCTAATCCCWTT
16S rRNA *Microcystis* sp.	Micr 184F	GCCGCRAGGTGAAAMCTAA
	Micr 431R	AATCCAAARACCTTCCTCCC
16S rDNA *Dolichospermun* sp.	Ana 573F	AGTGGAAACTACAAAGCTAGAGTT
	Ana 780R	CTTGGGTCGATACGAGCT
Microcystin *mcy A*	mcyA_CD1F	AAAATTAAAAGCCGTATCAAA
	mcyA_CD1R	AAAAGTGTTTTATTAGCGGCTCAT
Microcystis sp. *mcyA*	MSF	ATCCAGCAGTTGAGCAAGC
	MSR	TGCAGATAACTCCGCAGTTG
Microcystis sp. *mcyB*	2156-F	ATCACTTCAATCTAACGACT
	3111-R	AGTTGCTGCTGTAAGAAA
Microcystis sp. *mcyC*	PSCF1	GCAACATCCCAAGAGCAAAG
	PSCR1	CCGACAACATCACAAAGGC
Microcystis sp. *mcyD*	PKDF1	GACGCTCAAATGATGAAAC
	PKDR1	GCAACCGATAAAAACTCCC
Microcystis sp. *mcyE*	PKEF1	CGCAAACCCGATTTACAG
	PKER1	CCCCTACCATCTTCATCTTC
Cylindrospermopsins *cyrJ*	cynsulF	ACTTCTCTCCTTTCCCTATC
	cylnamR	GAGTGAAAATGCGTAGAACTTG
Saxitoxins *sxtl*	sxtl682F	GGATCTCAAAGAAGATGGCA
	sxtl877R	GCCAAACGCAGTACCACTT
Anatoxins *AnaC*	anaC-gen F	TCTGGTATTCAGTCCCCTCTAT
	anaC-gen R	CCCAATAGCCTGTCATCAA

Table S3. Cyanotoxins, parameters for MS analysis and operation and instrument limit of detection (LOD).

Cyanotoxin	RT (min)	MRM Transitions (*m/z*)	Cone (V)	Collision energy (eV)	LOD (μg/L)
Microcystin-LR	16.09	995.5>135<br>995.5>599	50<br>50	30<br>30	2.9 ± 0.8
Microcystin-RR	14.42	520>105<br>520>135	20<br>20	40<br>30	4.1 ± 0.3
Microcystin-YR	15.52	1045.5>135<br>1045.5>599	20<br>20	60<br>55	3.2 ± 0.8
Microcystin-LW	19.30	1047.3>579.5	50	20	4.6 ± 1.3
Microcystin-LA	18.41	932.5>135<br>932.5>599.1	50<br>50	30<br>30	2.9 ± 0.8
Microcystin-LF	20.41	1008.6>515.1	50	20	8.2 ± 2.8
Microcystin-LY	18.38	1024.5>677.2	50	20	13 ± 3.2
Nodularin R	15.42	825.3>135.5	50	20	4.2 ± 3.2
Cylindrospermopsin	4.43	416>176<br>416>336	20<br>20	80<br>20	3.8 ± 0.7
Anatoxin a	5.82	166>131<br>166>149	20<br>20	25<br>15	5.7 ± 0.3
Saxitoxin	3.24	300>125<br>300>204	20<br>20	25<br>25	7.9 ± 1.3

Table S4. Microcystins analyses conditions carried out by external laboratory CIFGA S.A.

Phase	Descriptions			
Equipment	HPLC pump	Waters ACQUITY UPLC System.		
	MS detector	Waters XEVO TQ-MS.		
	HPLC column	ACQUITY UPLC HSST3 2.1x100 mm, 1.7µm.		
	Software	MassLynx 4.1.		
HPLC conditions	HPLC mobile phase solution (A): Water containing 0.1 % (v/v) FmOH.			
	HPLC mobile phase solution (B): Acetonitrile containing 0.1 % (v/v) FmOH.			
	**Gradient UPLC conditions.**			
	**Time/min**	**Flow/mLmin^{-1}**	**%A**	**%B**
	0.0	0.3	70	30
	0.8	0.3	70	30
	2.5	0.3	40	60
	5.0	0.3	0	100
	5.5	0.3	0	100
	5.6	0.3	70	30
	7.6	0.3	70	30
	Blank:		methanol	
	Sample dilution:		methanol	
	Column temperature:		30 °C.	
	Sample chamber temperature:		8 °C	
	Injection volume:		5 µL	
LC-MS conditions	Capillary voltage:		2.5 kV	
	Desolvation nitrogen flow:		900 L/h	
	Desolvation temperature:		350 °C	
	Detector ionization mode:		ESI +	
	Adquisition:		MRM (Multiple Reaction Monitoring)	

Table S5. Field parameters in water samples from El Pañe reservoir. Temperaturre, pH and Conductivity. Field parameters in water samples from El Pañe reservoir. TDS: Total Dissolved Solids (TDS), Dissolved Oxygen (DO) and Turbidity. Field parameters in water samples from El Pañe reservoir. Total Phosphorus, Total Nitrogen and Chlorophyll A.

Code	Origin	Month	Results		
			Temperature (°C)	pH	Conductivity (μS/cm)
EPP1M	El Pañe	May	9.51	7.59	54
EPP2M	El Pañe	May	-	-	-
EPP3M	El Pañe	May	-	-	-
EPP1N	El Pañe	November	-	-	-
EPP2N	El Pañe	November	12.70	8.84	51
EPP3N	El Pañe	November	11.59	7.74	57

Code	Origin	Month	Results		
			TDS (ppm)	DO (mg/L)	Turbidity (FNU)
EPP1M	El Pañe	May	27	3.94	14.5
EPP2M	El Pañe	May	-	-	-
EPP3M	El Pañe	May	-	-	-
EPP1N	El Pañe	November	-	-	-
EPP2N	El Pañe	November	25	9.79	186
EPP3N	El Pañe	November	29	4.70	194

Code	Origin	Month	Results		
			Total Phosphorus (mg/L)	Total Nitrogen (mg/L)	Chlorophyll A (μg/L)
EPP1M	El Pañe	May	-	-	-
EPP2M	El Pañe	May	-	-	-
EPP3M	El Pañe	May	-	-	-
EPP1N	El Pañe	November	-	-	-
EPP2N	El Pañe	November	0.006	0.77	3.3
EPP3N	El Pañe	November	0.004	0.76	2.9

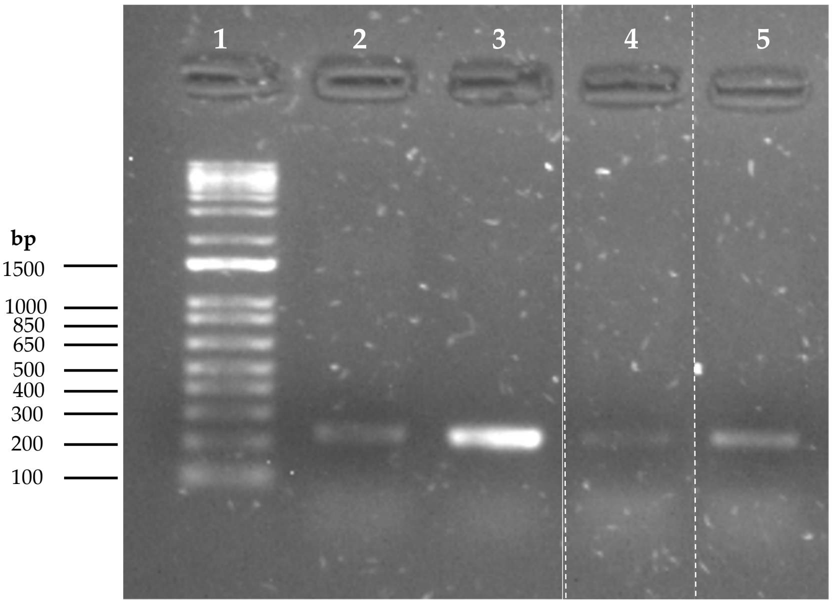

Figure S1. Amplification of 16S rRNA gene specific for *Microcystis* sp. in samples from El Pañe reservoir. The numbers showed represent: **1.** 1Kb Ladder, **2.** EPP3N, **3.** EPP2N, **4.** EPP3M, and **5.** EPP2M

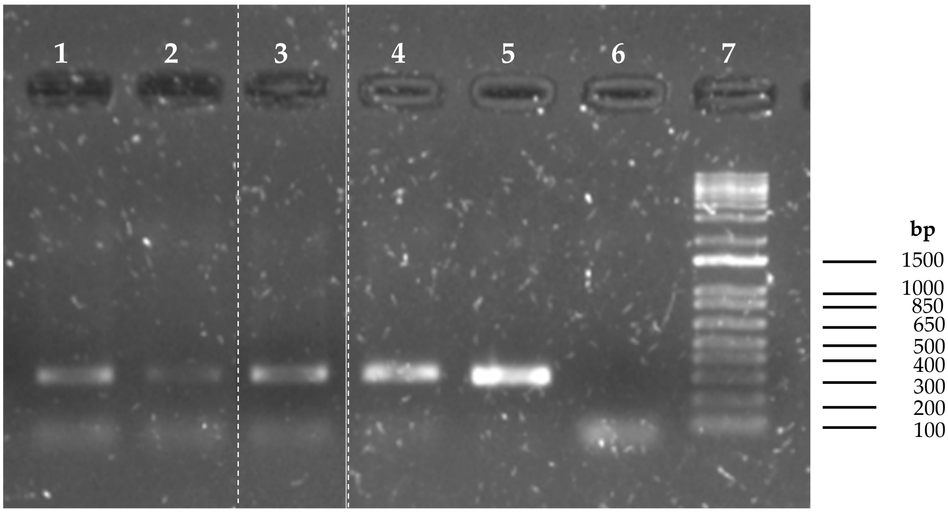

Figure S2. Amplification of microcystin encoding-gene (*mcyA*) in samples from El Pañe reservoir. The numbers showed represent: **1.** EPP2M, **2.** EPP2N, **3.** EPP3M, **4.** EPP3N, **5.** Positive Control, **6.** Negative Control, and **7.** 1Kb Ladder
